# Association of Adjuvant Chemotherapy With Overall Survival Among Patients With Locally Advanced Gastric Cancer After Neoadjuvant Chemotherapy

**DOI:** 10.1001/jamanetworkopen.2022.5557

**Published:** 2022-04-01

**Authors:** Jian-Xian Lin, Yi-Hui Tang, Guan-Jie Lin, Yu-Bin Ma, Jacopo Desiderio, Ping Li, Jian-Wei Xie, Jia-Bin Wang, Jun Lu, Qi-Yue Chen, Long-Long Cao, Mi Lin, Ru-Hong Tu, Chao-Hui Zheng, Amilcare Parisi, Mark J. Truty, Chang-Ming Huang

**Affiliations:** 1 Department of Gastric Surgery, Fujian Medical University Union Hospital, Fuzhou, Fujian Province, China; 2 Key Laboratory of Ministry of Education of Gastrointestinal Cancer, Fujian Medical University, Fuzhou, Fujian Province, China; 3 Department of Gastrointestinal Surgery, Qinghai University Affiliated Hospital, Xining, China; 4 Department of Digestive Surgery, St Mary’s Hospital, Terni, Italy; 5 Department of Surgical Sciences, La Sapienza University of Rome, Rome, Italy; 6 Section of Hepatobiliary and Pancreatic Surgery, Division of Subspecialty General Surgery, Department of Surgery, Mayo Clinic, Rochester, Minnesota

## Abstract

**Importance:**

Neoadjuvant chemotherapy (NAC) is a standard treatment option for locally advanced gastric cancer (LAGC); however, the indications for adjuvant chemotherapy (AC) in patients with LAGC who received NAC remain controversial.

**Objective:**

To compare survival rates between patients with LAGC who received AC and those who did not after NAC followed by surgery.

**Design, Setting, and Participants:**

This multicenter, international cohort study included 353 patients with LAGC undergoing curative-intent gastrectomy after NAC at 2 tertiary referral teaching hospitals in China between June 1, 2008, and December 31, 2017. To externally validate the findings in the Chinese patients, 109 patients from the US and Italy between June 1, 2006, and June 30, 2013, were reviewed. The follow-up period of the Chinese patients was completed in December 2020, and the follow-up period of the Western patients was completed between February and July 2017. Data analysis was performed from December 1, 2020, to February 28, 2021.

**Exposures:**

Patients who received AC and those who did not were propensity score matched to evaluate the association of AC with survival.

**Main Outcomes and Measures:**

Overall survival (OS), disease-free survival, and disease-specific survival.

**Results:**

Of 353 patients from China (275 [78.1%] male; mean [SD] age, 58.0 [10.7] years), 262 (74.1%) received AC and 91 (25.9%) did not. After propensity score matching, the 3-year OS was significantly higher in patients who received AC (60.1%; 95% CI, 53.1%-68.1%) than in those who did not (49.3%; 95% CI, 39.8%-61.0%) (*P* = .02). Lymph node ratio (LNR) was significantly associated with AC benefit (*P* < .001 for interaction), and a plot of the interaction between LNR and AC demonstrated that AC was associated with improved OS in patients with higher (≥9%) LNRs (3-year OS: 46.6% vs 21.7%; *P* < .001), but not in patients with LNRs less than 9% (3-year OS: 73.9% vs 71.3%; *P* = .30). When stratified by AC cycles, only those patients who completed at least 4 AC cycles exhibited a significant survival benefit in the 6-month (hazard ratio, 0.56; 95% CI, 0.33-0.96; *P* = .03) and 9-month landmark analysis (hazard ratio, 0.50; 95% CI, 0.27-0.94; *P* = .03). In the external cohort, improved OS with AC administration was also found in patients with LNRs of 9% or greater (3-year OS: 53.0% vs 26.3%; *P* = .04).

**Conclusions and Relevance:**

In this cohort study, the administration of AC after NAC and resection of LAGC was associated with improved prognosis in patients with LNRs of 9% or greater. These findings suggest that LNR might be valuable in AC selection in future decision-making processes.

## Key Points

**Question:**

Is adjuvant chemotherapy associated with a survival benefit for patients with locally advanced gastric cancer who underwent curative-intent gastrectomy after neoadjuvant chemotherapy?

**Findings:**

In this cohort study of 462 patients with locally advanced gastric cancer from Western and Eastern countries, adjuvant chemotherapy was associated with significantly improved survival in patients with a lymph node ratio of at least 9% compared with those who did not receive adjuvant chemotherapy.

**Meaning:**

These findings suggest that the lymph node ratio could be useful in adjuvant chemotherapy selection for locally advanced gastric cancer after neoadjuvant chemotherapy in future decision-making processes.

## Teaser

This cohort study compares survival among patients with locally advanced gastric cancer who did and did not receive adjuvant chemotherapy after neoadjuvant chemotherapy followed by surgery.

## Introduction

Gastric cancer is a global health problem, with more than 1 million newly diagnosed cases worldwide each year.^1,2^ Although improvements in surgical techniques and perioperative care have led to decreased surgical morbidity and mortality,^3^ the prognosis of patients diagnosed with advanced-stage gastric cancer remains poor, even after complete resection.^2,4^ More than half of patients died of disease recurrence.^5,6^ To overcome the limitations of surgery alone, multimodal treatments, such as perioperative chemotherapy or neoadjuvant chemotherapy (NAC), have been extensively examined in the past 20 years. The landmark MAGIC (Medical Research Council Adjuvant Gastric Infusional Chemotherapy) trial published in 2006 first demonstrated that perioperative chemotherapy with epirubicin, cisplatin, and fluorouracil or capecitabine (ECF/ECX) achieved a 13% improvement in overall survival (OS) compared with surgery alone.^7^ More recently, a large-scale German study^8,9^ clearly showed the superiority of neoadjuvant docetaxel, oxaliplatin, fluorouracil, and leucovorin (FLOT) over ECF/ECX in terms of pathological response and OS. Since then, perioperative chemotherapy has been widely used in the treatment of patients with locally advanced gastric cancer (LAGC).^10,11,12,13^

Although cumulative studies^7,9,14^ support the use of perioperative chemotherapy for LAGC, only 50% to 65% of patients who undergo NAC and surgery are able to start postoperative adjuvant chemotherapy (AC). More importantly, whether the receipt of AC can improve the outcome of patients with LAGC after NAC is still under debate. For example, van Putten et al^15^ demonstrated a better OS for patients who underwent perioperative treatment compared with those who underwent preoperative treatment only in a real-world study using the Netherlands Cancer Registry. However, in a more recent analysis^16^ using the National Cancer Database, the median OS was similar between patients who received AC and those who did not. Therefore, in the current study, we aimed to identify the indications for AC in patients with LAGC who underwent NAC and gastrectomy.

## Methods

### Study Population

In this cohort study, data from 353 consecutive patients with LAGC who underwent NAC and curative-intent gastrectomy between June 1, 2008, and December 31, 2017, at 2 institutions in China (183 patients from Fujian Medical University Union Hospital and 169 from Qinghai University Affiliated Hospital) were retrospectively reviewed. Moreover, we included an additional 109 patients from Western countries in the external validation cohort between June 1, 2006, and June 30, 2013 (88 patients from the Mayo Clinic and 21 from the International Study Group on Minimally Invasive Surgery for GASTRIC Cancer trial).^17^ We emulated the analyses used in the Capecitabine and Oxaliplatin Adjuvant Study in Stomach Cancer (CLASSIC) study,^18,19^ the most recently published randomized clinical trial to estimate the effect of AC on survival outcomes. We mirrored each protocol component as closely as possible, with several modifications to accommodate our use of retrospective data (eTable 1 in the Supplement). The flow diagram is shown in eFigure 1 in the Supplement. All patients were divided into the AC and non-AC groups. All procedures followed were in accordance with the ethical standards of the responsible committee on human experimentation (institutional and national) and with the Declaration of Helsinki.^20^ Written informed consent was obtained from all patients for being included in the study. The study was approved by the institutional review boards of each participating institution. This study followed the Strengthening the Reporting of Observational Studies in Epidemiology (STROBE) reporting guideline.^21^

### Perioperative Chemotherapy and Surgery

All patients received fluorouracil-based NAC and were routinely recommended to receive AC after surgery, including 2 preoperative and 6 postoperative 3-week cycles of SOX/XELOX (40 to 60 mg/m^2^ of S-1 or 1000 mg/m^2^ of capecitabine orally twice daily on days 1 to 14 and 130 mg/m^2^ of oxaliplatin intravenously on day 1),^22^ 3 preoperative and 3 postoperative 3-week cycles of ECF/ECX (50 mg/m^2^ of epirubicin intravenously on day 1), and 3 preoperative and 6 postoperative 2-week cycles of FOLFOX4 (85 mg/m^2^ of oxaliplatin intravenously on day 1, 200 mg/m^2^ of folinic acid as a 2-hour intravenous infusion followed by a 400-mg/m^2^ bolus of fluorouracil, and a 22-hour intravenous infusion of 600 mg/m^2^ of fluorouracil).^23^ The regimens and doses of the perioperative chemotherapy were planned by professional oncologists at each institution and would be adjusted according to drug toxic effects or tumor responses. Surgery was performed 3 weeks after the NAC. All surgical procedures, including D2 lymph node dissection, were performed according to the guidelines of the Japanese Research Society for the Study of Gastric Cancer,^24,25^ whereas staging was performed according to the ypTNM classification.^26^

### Definitions

Patients who underwent 1 or more postoperative AC cycles within 3 months after surgery constituted the AC group. Patients who did not undergo postoperative AC and those who did not initiate AC within 3 months constituted the non-AC group. Pretreatment comorbidities were assessed using the Charlson-Deyo Comorbidity Index.^27^ The neutrophil-to-lymphocyte ratio was calculated by dividing the neutrophil count by the lymphocyte count.^28^ The lymphocyte-to-monocyte ratio was calculated by dividing the lymphocyte count by the monocyte count.^29^ The prognostic nutritional index was calculated as follows: 10 × serum albumin (grams per liter) + 0.005 × lymphocyte count (cubic millimeters).^30^ The lymph node ratio (LNR) is defined as the number of positive lymph nodes divided by the number of examined lymph nodes. Pathological response was quantified using the Becker regression criteria.^31^ For all survival analyses, survival time was defined as time from 3 months after surgery to death or recurrence or to the last follow-up.

### Follow-up Investigation

All patients were followed up postoperatively every 3 months for 2 years, every 6 months during years 3 to 5, and annually thereafter, with physical examinations, laboratory tests (including carcinoembryonic antigen and cancer antigen 19-9), and imaging examinations (including chest radiography or chest computed tomography, abdominal ultrasonography, or abdominopelvic computed tomography). In addition, annual endoscopy was recommended. The follow-up period of the Chinese patients was completed in December 2020, with a median follow-up time of 32 months (range, 3-135 months). The follow-up period of the Western patients was completed between February and July 2017, with a median follow-up time of 32 months (range, 3-121 months).

### Statistical Analysis

The primary end point of this study was OS. Secondary end points included disease-free survival (DFS) and disease-specific survival (DSS). Continuous variables are presented as means (SDs) or medians (IQRs), and categorical variables were presented as numbers and percentages. Categorical variables were assessed using the χ^2^ test or the Fisher exact test, and continuous variables were compared using the 2-tailed, unpaired *t* test. Unadjusted survival was assessed using Kaplan-Meier estimates, and the differences were assessed using log-rank tests. Univariable and multivariable analyses were performed using the Cox proportional hazards regression model to investigate the association between clinicopathological parameters and OS, DFS, and DSS using the survival package of R software. Owing to the matching procedure, we used robust SEs to adjust for the correlation between matched pairs in the Cox proportional hazards regression model.^32^ Because multiple outcomes were assessed, *P* values were adjusted by controlling for the false discovery rate for tests of interaction and multivariable analyses using the Benjamini-Hochberg procedure.^33^ The false discovery rate 2-sided *P* value was controlled at .05, which means that less than 5% of the declared significant test results can be expected to be false positives.

The propensity score method was used to minimize the potential bias caused by confounding covariates using the MatchIt package of R software. A logistic regression model was constructed to generate propensity scores. The clinicopathological factors included in the model were age, sex, comorbidities, Lauren classification, type of gastrectomy, tumor location, ypT and ypN stage, LNR, lymphovascular and neural invasion, R status, postoperative complications, time to ambulation, and postoperative hospital stay in the Eastern cohort. In the Western cohort, patients were matched based on region, age, sex, American Society of Anesthesiologists score, histologic type, type of gastrectomy, lymph node dissection, tumor location, ypT and ypN stage, LNR, R status, and postoperative complications. To maximize the number of participants, patients who received AC were matched to those who did not in the Eastern (Western) cohort at a 2:1 (1:1) ratio, using a “greedy” nearest-neighbor matching algorithm with no replacement.^34^ Baseline characteristics between the propensity score–matched groups were compared using *P* values.

The heterogeneous effect of AC on OS in different subgroups was assessed by creating interaction terms between each clinicopathological parameter and AC in the Cox proportional hazards regression model. All interaction terms were fitted to a separate model, adjusted for potential confounders. A statistically significant interaction term indicates that the effect of AC on OS differs depending on the value of the specified covariate.^35^ Moreover, the associations between LNR and OS in patients who did or did not receive AC were modeled using restricted cubic splines (RCSs) using the rms package of R software.^36^ Because there was no obvious inflection point to represent a potential threshold in the RCS model, the optimal threshold was defined as the value above which the patients could achieve maximum survival benefit from AC, by using maximally selected rank statistics (maxstat package).^37^ Additional subgroup analyses were also performed.

To investigate the association of AC cycles with survival benefits, we applied a landmark approach to account for immortal time bias.^15,38,39,40^ Survival outcomes were compared between patients who completed at least 4 AC cycles and those who completed 1 to 3 AC cycles. Most patients who completed at least 4 AC cycles initiated the fourth cycle within 6 months and no later than 9 months after surgery. Thus, patients who experienced death or disease recurrence within 6 or 9 months after surgery were excluded from the 6-month and 9-month landmark analysis. Statistical analysis was performed using SPSS, version 22.0 (SPSS Inc) and R software, version 4.0.3 (R Foundation for Statistical Computing). Data analysis was performed from December 1, 2020, to February 28, 2021.

## Results

### Baseline Characteristics

Between June 2008 and December 2017, a total of 353 consecutive patients with LAGC who underwent NAC and curative-intent gastrectomy at the Fujian Medical University Union Hospital and Qinghai University Affiliated Hospital were included in this study. The baseline characteristics are summarized in Table 1. Of the included patients, 275 (78.1%) were men and 77 (21.9%) were women. The mean (SD) age at diagnosis was 58.0 (10.7) years. Most of the patients (262 [74.1%]) received postoperative AC (median, 3 cycles; range, 1-8 cycles). Patients who received AC were more likely to be younger (mean [SD] age, 57.3 [10.1] vs 60.1 [12.0] years; *P* = .03), have fewer comorbidities (Charlson-Deyo score of 0: 149 [57.1%] vs 38 [41.8%]; *P* = .04), have a shorter time to ambulation (median [IQR], 2 [1-2] vs 2 [2-2] days; *P* = .03), and have a shorter hospital stay (median [IQR], 13 [10-16] vs 11 [9-14] days; *P* = .01) than those who did not receive AC. After propensity score matching, no significant differences were found in baseline characteristics between the AC and non-AC groups (Table 1 and eFigure 2A in the Supplement).

**Table 1.** Baseline Characteristics of Study Patients Stratified by Receipt of Adjuvant Chemotherapy Before and After PSM in the Eastern Cohort^a^

Characteristic	Overall (N = 352)	Before PSM			After PSM	
		Non-AC (n = 91)	AC (n = 261)	*P* value	AC (n = 182), No. (%)	*P* value
Clinicopathological characteristics						
Age, mean (SD), y	58.0 (10.7)	60.1 (12.0)	57.3 (10.1)	.03	59.1 (10.1)	.48
Sex						
Male	275 (78.1)	71 (78.0)	204 (78.2)	.98	142 (78.0)	.99
Female	77 (21.9)	20 (22.0)	57 (21.8)		40 (22.0)	
Comorbidities						
0	187 (53.1)	38 (41.8)	149 (57.1)	.04	85 (46.7)	.74
1	124 (35.2)	39 (42.9)	85 (32.6)		72 (39.6)	
≥2	41 (11.6)	14 (15.4)	27 (10.3)		25 (13.7)	
Lauren classification						
Intestinal	109 (31.0)	30 (33.0)	79 (30.3)	.63	59 (32.4)	.93
Diffuse	243 (69.0)	61 (67.0)	182 (69.7)		123 (67.6)	
Type of gastrectomy						
Total	196 (55.7)	53 (58.2)	143 (54.8)	.62	108 (59.3)	.86
Distal	94 (26.7)	25 (27.5)	69 (26.4)		45 (24.7)	
Proximal	62 (17.6)	13 (14.3)	49 (18.8)		29 (15.9)	
Tumor location						
Lower third	84 (23.9)	19 (20.9)	65 (24.9)	.76	38 (20.8)	.98
Middle third	101 (28.7)	25 (27.5)	76 (29.1)		54 (29.7)	
Upper third	145 (41.2)	40 (44.0)	105 (40.2)		77 (42.3)	
Mixed	22 (6.3)	7 (7.7)	15 (5.7)		13 (7.1)	
ypT stage						
T0	10 (2.8)	2 (2.2)	8 (3.1)	.76	6 (3.3)	.96
T1	18 (5.1)	6 (6.6)	12 (4.6)		10 (5.5)	
T2	65 (18.5)	20 (22.0)	45 (17.2)		37 (20.3)	
T3	101 (28.7)	25 (27.5)	76 (29.1)		55 (30.2)	
T4	158 (44.9)	38 (41.8)	120 (46.0)		74 (40.7)	
ypN stage						
N0	117 (33.2)	37 (40.7)	80 (30.7)	.38	67 (36.8)	.90
N1	57 (16.2)	13 (14.3)	44 (16.9)		31 (17.0)	
N2	80 (22.7)	19 (20.9)	61 (23.4)		41 (22.5)	
N3	98 (27.8)	22 (24.2)	76 (29.1)		43 (23.6)	
Lymph node metastasis, median (IQR)	3 (0-7)	1 (0-6)	3 (0-8)	.24	2 (0-6)	.80
Lymph node harvested, median (IQR)	27 (19-36)	26 (18-39)	28 (20-36)	.87	28 (21-35)	.98
Lymph node ratio, median (IQR)	0.11 (0-0.27)	0.06 (0-0.24)	0.12 (0-0.28)	.22	0.09 (0-0.23)	.83
Lymphovascular invasion						
No	206 (58.5)	54 (59.3)	152 (58.2)	.85	107 (58.8)	.93
Yes	146 (41.5)	37 (40.7)	109 (41.8)		75 (41.2)	
Neural invasion						
No	203 (57.7)	55 (60.4)	148 (56.7)	.54	111 (61.0)	.93
Yes	149 (42.3)	36 (39.6)	113 (43.3)		71 (39.0)	
R status						
R0	317 (90.1)	86 (94.5)	231 (88.5)	.10	173 (95.1)	.85
R1	35 (9.9)	5 (5.5)	30 (11.5)		9 (4.9)	
Pathologic response						
TRG 1a/1b	47 (13.4)	12 (13.2)	35 (13.4)	.63	29 (15.9)	.29
TRG 2	151 (42.9)	42 (46.2)	109 (41.8)		70 (38.5)	
TRG 3	103 (29.3)	22 (24.2)	81 (31.0)		61 (33.5)	
Unknown	51 (14.5)	15 (16.5)	36 (13.8)		22 (12.1)	
Adjuvant chemotherapy cycle						
1-3	NA	NA	156 (59.5)	NA	107 (58.8)	NA
≥4	NA	NA	105 (40.5)		75 (41.2)	
Surgical outcomes, median (IQR)						
Blood loss, mL	100 (50-200)	100 (50-200)	100 (50-200)	.62	100 (50-200)	.63
Time to ambulation, d	2 (1-2)	2 (2-2)	2 (1-2)	.03	2 (1-2)	.11
Time to first flatus, d	3 (3-4)	3 (3-4)	3 (3-4)	.76	3 (3-4)	.53
Time to first liquid intake, d	5 (4-6)	5 (4-6)	5 (4-6)	.96	5 (4-6)	.84
Drainage tube removed time, d	9 (8-11)	9 (8-11)	9 (8-11)	.87	9 (8-11)	.86
Postoperative hospital stay, d	12 (9-14)	13 (10-16)	11 (9-14)	.01	12 (10-15)	.11
Postoperative complication						
No	270 (76.7)	69 (75.8)	201 (77.0)	.82	138 (75.8)	.99
Yes	82 (23.3)	22 (24.2)	60 (23.0)		44 (24.2)	
Immunologic and nutritional status (preoperative), median (IQR)						
Neutrophil to lymphocyte ratio	1.7 (1.2-2.7)	1.8 (1.2-2.7)	1.7 (1.2-2.7)	.98	1.8 (1.2-2.8)	.70
Lymphocyte to monocyte ratio	3.3 (2.6-5.1)	3.2 (2.6-4.5)	3.5 (2.6-5.1)	.58	3.4 (2.6-5.0)	.65
Prognostic nutritional index	47.0 (42.7-51.1)	46.5 (42.2-50.7)	47.1 (43.1-51.2)	.66	47.0 (42.7-50.8)	.99

Abbreviations: AC, adjuvant chemotherapy; NA, not applicable; PSM, propensity score matching; TRG, tumor regression grade.

^a^ Data are presented as number (percentage) of patients unless otherwise indicated.

eTable 1 in the Supplement details the baseline characteristics of the 109 patients in the Western cohort. Of these patients, 74 (67.9%) were men and 35 (32.1%) were women. The mean (SD) age at diagnosis was 61 (12.8) years. Seventy-four patients (67.9%) received AC, while the remaining 35 (32.1%) did not receive any further treatment after surgery. Undifferentiated tumors (28 [80.0%]; *P* = .004) and postoperative complications (11 [31.4%]; *P* = .03) were associated with the nonadministration of AC. After propensity score matching, no significant differences were found in the clinicopathologic characteristics between the 2 groups (eTable 2 and eFigure 2B in the Supplement).

### Overall Survival

Before matching, the 3-year OS was 55.2% (95% CI, 49.3%-61.9%) for those who received AC and 49.3% (95% CI, 39.3%-61.0%) for those who did not (*P* = .17) (eFigure 3A in the Supplement) in the Eastern cohort. After matching, the 3-year OS was significantly higher in patients who received AC (60.1%; 95% CI, 53.1%-68.1%) than in those who did not (49.3%; 95% CI, 39.8%-61.0%; *P* = .02) (eFigure 3B in the Supplement). In the multivariate analysis, the receipt of AC was independently associated with improved survival (OS: hazard ratio [HR], 0.56; 95% CI, 0.40-0.79; *P* = .007; DFS: HR, 0.58; 95% CI, 0.41-0.83; *P* = .03; DSS: HR, 0.55; 95% CI, 0.38-0.79; *P* = .007) (eTable 3 in the Supplement). In the Western cohort, a significant difference was found in OS between the AC and non-AC groups before matching (3-year OS: 64.8% vs 39.5%; *P* = .004); however, this difference did not reach statistical significance between the 2 groups after matching (3-year OS: 57.3% vs 39.5%; *P* = .11) (eFigure 3C and D in the Supplement).

### Association of LNR With AC Benefits

To further identify the subgroups of patients more likely to benefit from AC, we performed a stratified survival analysis in the matched Eastern cohort (Table 2). Among the clinicopathological characteristics, only LNR had a significant interaction with AC (*P* < .001 for interaction), with patients with higher LNR more likely to benefit from AC.

**Table 2.** Substratified Analysis for Overall Survival in the Matched Eastern Cohort

Variable	Hazard ratio (95% CI)	*P* value	FDR-adjusted *P* value for interaction
Age, y			
<60	0.70 (0.42-1.17)	.17	.38
≥60	0.60 (0.37-0.98)	.04	
Sex			
Male	0.69 (0.48-1.00)	.06	.43
Female	0.51 (0.23-1.12)	.09	
Comorbidities			
0-1	0.60 (0.41-0.89)	.01	.61
≥2	0.94 (0.43-2.03)	.87	
Lauren classification			
Intestinal	0.44 (0.22-0.88)	.02	.47
Diffuse	0.76 (0.50-1.14)	.18	
Tumor location			
Lower third	0.77 (0.34-1.76)	.54	.57
Middle third	0.68 (0.38-1.20)	.18	
Upper third	0.57 (0.31-1.05)	.07	
Mixed	0.59 (0.22-1.63)	.31	
ypT stage			
T0-1	0.52 (0.04-7.07)	.62	.97
T2	1.00 (0.45-2.23)	.99	
T3	0.30 (0.17-0.54)	<.001	
T4	0.79 (0.46-1.36)	.39	
ypN stage			
N0	0.79 (0.38-1.65)	.54	.07
N1	0.53 (0.19-1.47)	.22	
N2	0.45 (0.24-0.86)	.02	
N3	0.59 (0.33-1.05)	.07	
Lymph node harvested			
<15	0.74 (0.27-2.05)	.57	.77
≥15	0.63 (0.43-0.91)	.02	
Lymphovascular invasion			
No	0.73 (0.43-1.23)	.24	.66
Yes	0.55 (0.33-0.90)	.02	
Neural invasion			
No	0.68 (0.43-1.10)	.12	.93
Yes	0.61 (0.36-1.03)	.07	
R status			
R0	0.61 (0.42-0.88)	.009	.31
R1	1.31 (0.32-5.36)	.71	
Tumor regression^a^			
<50%	0.47 (0.23-0.94)	.03	.16
≥50%	0.80 (0.50-1.27)	.34	
Postoperative complication			
No	0.62 (0.40-0.96)	.03	.85
Yes	0.70 (0.37-1.30)	.28	

Abbreviation: FDR, false discovery rate.

^a^ Thirty-seven patients with missing data were excluded from survival analysis. Interaction terms between clinicopathological characteristics and adjuvant chemotherapy were included.

**Figure 1.**
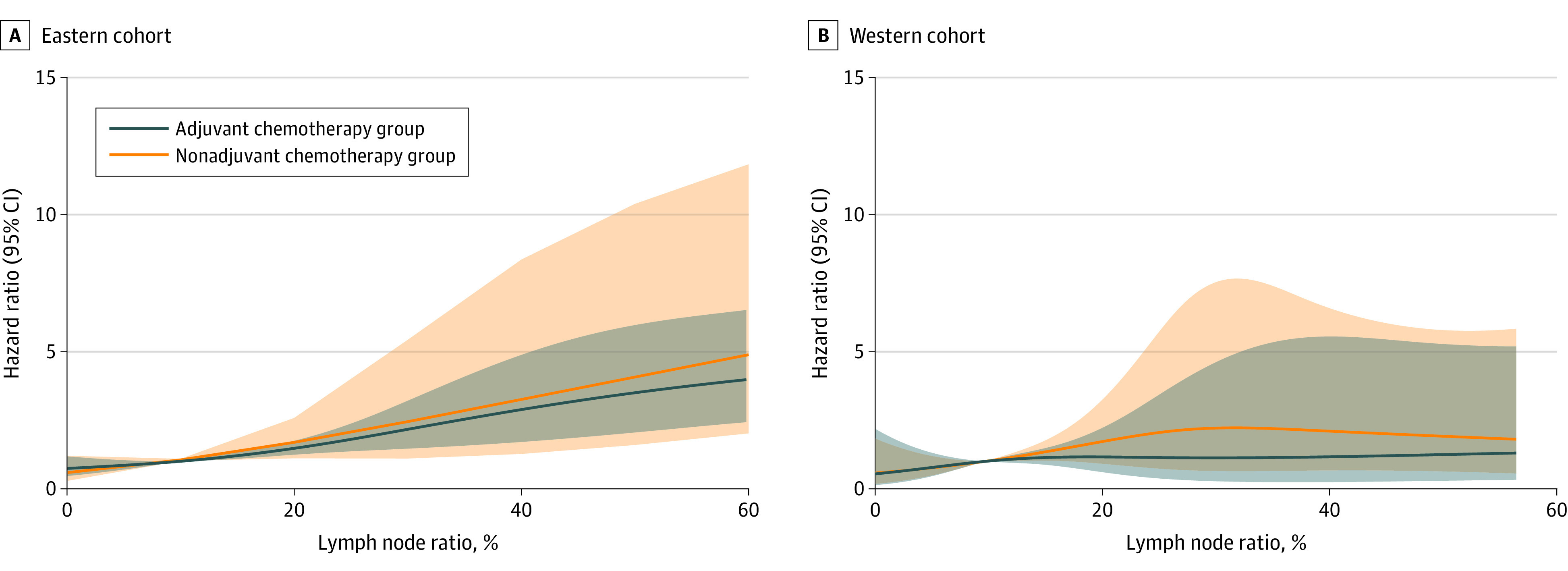
Interaction Among Lymph Node Ratio, Receipt of Adjuvant Chemotherapy, and Adjusted Hazard of Mortality Shaded regions represent the 95% CIs.

Figure 1 depicts a plot of the interaction between the LNR and AC modeled using RCS. The results revealed that patients with higher LNRs were more likely to experience a survival benefit from AC. Because there was no obvious inflection point to represent a potential threshold in the RCS model, the threshold above which the patients could achieve maximum survival benefit from AC was determined next in the matched Eastern cohort (optimal cutoff value of 9%) (eFigure 4 in the Supplement).

### Subgroup Analyses

**Figure 2.**
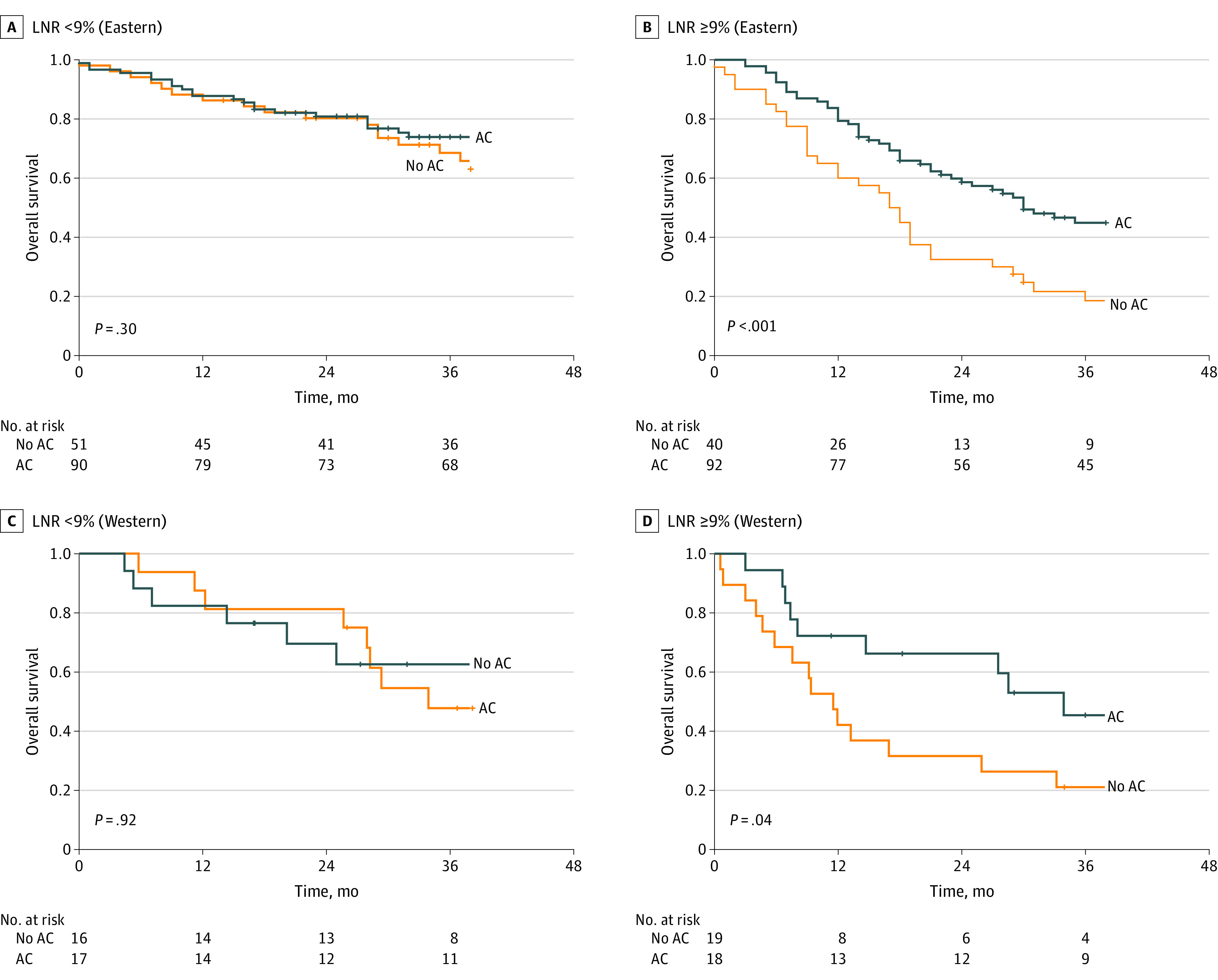
Kaplan-Meier Curves for Overall Survival Stratified by Lymph Node Ratio (LNR) in the Eastern and Western Cohorts After Propensity Score Matching *P* values were calculated using the log-rank test. The time zero was set as 3 months after surgery. AC indicates adjuvant chemotherapy.

A subgroup analysis divided patients into those with an LNR less than 9% and those with an LNR of 9% or greater in the matched cohorts. In the Eastern cohort, in 141 patients with an LNR less than 9%, the receipt of AC was not associated with improved survival compared with those who did not receive AC (Figure 2A and eFigure 5A in the Supplement).

In 132 patients with an LNR of 9% or greater, the receipt of AC was significantly associated with improved survival compared with no AC (3-year OS: 46.6% vs 21.7%, *P* < .001; 3-year DFS: 49.5% vs 20.0%, *P* < .001; 3-year DSS: 51.1% vs 22.5%, *P* < .001) (Figure 2B and eFigure 5B in the Supplement). In the multivariate analysis, AC was associated with a survival benefit in patients with an LNR of 9% or greater (OS: HR, 0.45; 95% CI, 0.29-0.69; *P* = .007; DFS: HR, 0.47; 95% CI, 0.30-0.73; *P* = .008; DSS: HR, 0.47; 95% CI, 0.30-0.72; *P* = .007) (Table 3) but was not associated with a survival benefit in those with an LNR less than 9% (eTable 4 in the Supplement). In the Western cohort, AC was associated with improved OS in patients with an LNR of 9% or greater (3-year OS: 53.0% vs 26.3%; *P* = .04; hazard ratio [HR], 0.43; 95% CI, 0.21-0.89; *P* = .02) but not with an LNR less than 9% (3-year OS: 62.6% vs 54.5%, *P* = .92; HR, 0.95; 95% CI, 0.38-2.38; *P* = .91) (Figure 2C and D and eTable 5 in the Supplement).

**Table 3.** Univariable and Multivariable Analyses for Overall Survival, Disease-Free Survival, and Disease-Specific Survival in Patients With a Lymph Node Ratio of 9% or Greater in the Matched Eastern Cohort

Variable	Overall survival, univariate analysis		Multivariate analysis								
			Overall survival			Disease-free survival			Disease-specific survival		
	HR (95% CI)	*P* value	HR (95% CI)	*P* value^a^	HR (95% CI)		*P* value^a^	HR (95% CI)		*P* value^a^
Age	1.00 (0.99-1.02)	.85	NA	NA	NA		NA	NA		NA
Sex										
Male	1 [Reference]	.60	NA	NA	NA		NA	NA		NA
Female	1.16 (0.67-2.00)		NA		NA			NA		
Comorbidities										
0-1	1 [Reference]	.15	NA	NA	NA		NA	NA		NA
≥2	1.24 (0.92-1.68)		NA		NA			NA		
Lauren classification										
Intestinal	1 [Reference]	.96	NA	NA	NA		NA	NA		NA
Diffuse	1.01 (0.65-1.57)		NA		NA			NA		
Tumor location										
Lower third	1 [Reference]	.14	NA	NA	NA		NA	NA		NA
Middle third	1.64 (0.81-3.32)		NA		NA			NA		
Upper third	1.66 (0.81-3.40)		NA		NA			NA		
Mixed	2.18 (0.75-6.38)		NA		NA			NA		
ypT stage										
T1-2	1 [Reference]	.23	NA	NA	NA		NA	NA		NA
T3	0.97 (0.50-1.87)		NA		NA			NA		
T4	1.40 (0.76-2.58)		NA		NA			NA		
ypN stage										
N1	1 [Reference]	<.001	1 [Reference]	.008	1 [Reference]		.03	1 [Reference]		.007
N2	1.97 (0.84-4.59)		1.92 (0.85-4.37)		2.30 (0.78-6.73)			3.08 (1.07-8.86)		
N3	3.64 (1.64-8.11)		3.21 (1.48-6.94)		3.58 (1.18-10.83)			5.15 (1.78-14.86)		
Lymphovascular invasion										
No	1 [Reference]	.01	1 [Reference]	.07	1 [Reference]		.03	1 [Reference]		.05
Yes	1.63 (1.11-2.39)		1.61 (1.09-2.38)		1.76 (1.20-2.58)			1.72 (1.16-2.57)		
Neural invasion										
No	1 [Reference]	.42	NA	NA	NA		NA	NA		NA
Yes	1.18 (0.79-1.78)		NA		NA			NA		
R status										
R0	1 [Reference]	.007	1 [Reference]	.07	1 [Reference]		.50	1 [Reference]		.11
R1	2.35 (1.26-4.38)		2.32 (1.17-4.61)		1.47 (0.75-2.89)			2.12 (1.06-4.24)		
Tumor regression^b^										
<50%	1 [Reference]	.89	NA	NA	NA		NA	NA		NA
≥50%	1.03 (0.64-1.67)		NA		NA			NA		
Postoperative complication										
No	1 [Reference]	.33	NA	NA	NA		NA	NA		NA
Yes	0.79 (0.49-1.27)		NA		NA			NA		
Adjuvant chemotherapy										
No	1 [Reference]	<.001	1 [Reference]	.007	1 [Reference]		.008	1 [Reference]		.007
Yes	0.46 (0.29-0.71)		0.45 (0.29-0.69)		0.47 (0.30-0.73)			0.46 (0.30-0.72)		

Abbreviations: FDR, false discovery rate; HR, hazard ratio; NA, not applicable (variable not included in the multivariate analysis).

^a^ FDR-adjusted *P* value.

^b^ Twenty patients with missed data were excluded from survival analysis.

Next, we performed a stratified survival analysis for patients with an LNR less than 9% and those with an LNR of 9% or greater in the Eastern cohort. In patients with an LNR less than 9%, no subgroups of patients had a survival benefit from AC (eTable 6 in the Supplement). In patients with an LNR of 9% or greater, only ypT stage had a significant association with AC (*P* = .03 for interaction). Patients with T3-4 disease (T3: HR, 0.18; 95% CI, 0.08-0.40; *P* < .001; T4: HR, 0.49; 95% CI, 0.28-0.88; *P* = .02) were more likely to benefit from AC (eTable 7 in the Supplement).

### Association of AC Cycles With Survival Benefits

The landmark analysis (chosen as 6 and 9 months postoperatively, with sample sizes of 108 and 98 patients, respectively) was performed to investigate the association of AC cycles with survival benefits. The results revealed that in patients with an LNR of 9% or greater, the survival rates were significantly better in patients who completed at least 4 AC cycles than in those who did not receive AC (6-month landmark: HR, 0.56; 95% CI, 0.33-0.96; *P* = .03; 9-month landmark: HR, 0.50; 95% CI, 0.27-0.94; *P* = .03). However, no survival difference was found between those who completed only 1 to 3 AC cycles and those who did not receive AC (eFigure 6 in the Supplement).

## Discussion

To the best of our knowledge, this is the first multicenter, international cohort study demonstrating a meaningful association between LNR and the survival benefit associated with AC in patients with LAGC who underwent curative-intent gastrectomy after NAC. The receipt of AC was associated with improved OS, DFS, and DSS in patients with an LNR of 9% or greater but was not associated with a survival benefit in patients with an LNR of less than 9%. Moreover, in the subgroup of patients with an LNR of 9% or greater, those with T3-4 disease and those who completed at least 4 AC cycles were more likely to achieve a survival benefit. These results suggest that LNR may be the most important factor associated with the benefits of AC.

Perioperative chemotherapy has been widely recommended as a standard treatment for LAGC in both Western and Eastern countries.^10,11,12,13^ This concept is based on the positive results of 2 benchmark phase 3 trials: the MAGIC^7^ and the Fédération Nationale des Centres de Lutte contre le Cancer/Fédération Francophone de Cancérologie Digestive (FNCLCC/FFCD) trials.^14^ In both of these prospective randomized clinical trials,^7,14^ patients were 1:1 randomly assigned to perioperative chemotherapy followed by surgical resection or to surgical resection alone. The 5-year OS was significantly improved by 13% in the chemotherapy and surgical resection group and 14% in the surgical resection alone group after treatment with perioperative chemotherapy plus surgery. However, the scheduled postoperative chemotherapy was delivered to 66% of patients in the MAGIC trial^7^ and 50% of patients in the FNCLCC/FFCD trial.^14^ Therefore, both trials failed to demonstrate the role of the postoperative component in the survival benefit of perioperative chemotherapy. In contrast, another randomized clinical trial of NAC without administration of any postoperative treatment was not able to reproduce the OS benefit in LAGC.^41^ This finding indicates that the administration of AC may provide a survival benefit after NAC followed by surgery.

The designated postoperative chemotherapy is reportedly omitted in nearly half of the patients because of postoperative complications, poor nutritional status, functional decline, and residual toxicity from preoperative therapy.^7,9,14,42^ More importantly, it is still under debate whether the receipt of AC was associated with improved OS in patients with LAGC who underwent NAC and subsequent gastrectomy. In 66 patients treated with perioperative ECF chemotherapy for gastric and gastroesophageal junction adenocarcinomas, patients who received both NAC and AC had better outcomes compared with those who only received NAC.^43^ A larger study^44^ of 134 patients treated with perioperative ECF, ECX, or FLOT revealed that administration of AC may contribute to the achieved survival benefit, especially in the presence of lymph node metastasis and poor histologic regression. In contrast, Drake et al^16^ demonstrated contradictory results: the influence of AC on OS appeared to be limited. Another prospective study^45^ is investigating whether strategies that use all multimodality treatment before surgery will improve cancer-related outcomes. However, none of these studies examined the association of LNR with the potential benefit of AC in such patients. In the current study, we demonstrated a significant interaction between LNR and AC: the receipt of AC was associated with improved survival in patients with an LNR of 9% or greater but was not associated with a survival benefit in patients with an LNR less than 9%. One possible explanation is that patients with a high LNR are at high risk of developing tumor recurrence after radical gastrectomy,^46,47^ which necessitates the focus on effective systemic elimination of micrometastases, which can be achieved by the addition of AC.^48^

In addition to the beneficial effect of AC on survival, we should also determine the appropriate number of AC cycles. In most previous studies,^49,50^ performing a sufficient number of AC cycles could improve oncologic outcomes for advanced gastric cancer. In a retrospective study of 385 patients, Luc et al^51^ also demonstrated that at least 2 cycles of AC were necessary to improve survival in patients with gastroesophageal adenocarcinoma. In the current study, the analysis revealed that in patients with higher LNR, 3-year survival was significantly higher in patients who completed at least 4 AC cycles than in those who completed 1 to 3 AC cycles for the Eastern cohort. Moreover, no survival difference was found between those who completed only 1 to 3 AC cycles and those who did not receive AC. Therefore, performing at least 4 AC cycles may be associated with improved prognosis.

### Limitations

Our study has some limitations that need to be acknowledged. First, as a retrospective study, our results may have been subject to selection bias. Although propensity score matching was performed to minimize such bias, the groups may not have been completely comparable, and confounding because of the nonrandomized assignment of treatment may still have occurred. Moreover, it is unrealistic to perform an intent-to-treat comparison between intervention and controlled groups, which will introduce immortal time bias. Although the landmark approach controlled for immortal time bias by limiting the analysis to patients who survived 6 or 9 months, it may suffer a major loss of statistical efficiency and precision.^52^ The current study also excluded patients who died within 3 postoperative months, which resulted in limited generalizability. Second, we included patients who underwent various perioperative chemotherapy schemes, including a diversity in the preplanned number of neoadjuvant and adjuvant cycles; the effect of different schemes on survival should not be neglected. Third, patients, together with the care delivered in the 2 tertiary referral teaching hospitals, may not be a good representation of the general Chinese patient population or the general care delivered in China. In addition, the sample size of the Western cohort was not large, which limited validity. Nevertheless, significant associations between LNR and AC benefit were found in both cohorts with heterogeneous characteristics, which to some extent validates the generalizability of our findings. Fourth, the results in the subgroup analysis based on the 9% cutoff for LNR in the Eastern cohort may be optimistic because the same data have been used twice. We will continue following up the study patients and update the results in the future. Furthermore, a large, multicenter randomized clinical trial may be needed to confirm the findings.

## Conclusions

In this multicenter, international cohort study of patients with LAGC who underwent NAC followed by curative-intent gastrectomy, the administration of AC was not associated with a survival benefit in patients with an LNR less than 9%, whereas patients with an LNR of 9% or greater experienced a significant survival benefit. Lymph node ratio may be used as an adjunct in clinical decision-making regarding AC planning in this patient population. Additional studies are needed to confirm these findings.

## Notes

Supplement. **eTable 1.** Specification and Emulation of a Target Trial **eTable 2.** Baseline Characteristics of Study Patients Stratified by Receipt of Adjuvant Chemotherapy Before and After Propensity Score Matching in the Western Cohort **eTable 3.** Univariable and Multivariable Analyses for Overall Survival, Disease-Free Survival, and Disease-Specific Survival in the Matched Eastern Cohort **eTable 4.** Univariable and Multivariable Analyses for Overall Survival, Disease-Free Survival, and Disease-Specific Survival in Patients With a Lymph Node Ratio <9% in the Matched Eastern Cohort **eTable 5.** Univariable Analyses for Overall Survival in the Matched Western Cohort **eTable 6.** Substratified Analysis for Overall Survival, Disease-Free Survival, and Disease-Specific Survival in Patients With a Lymph Node Ratio <9% in the Matched Eastern Cohort **eTable 7.** Substratified Analysis for Overall Survival, Disease-Free Survival, and Disease-Specific Survival in Patients With a Lymph Node Ratio ≥9% in the Matched Eastern Cohort **eFigure 1.** Diagram of Study Population in the Eastern (A) and Western (B) Cohorts **eFigure 2.** The Distribution of Propensity Scores Before and After Matching in the Eastern (A) and Western (B) Cohorts **eFigure 3.** Kaplan-Meier Curves for Overall Survival (Disease-Free Survival, Disease-Specific Survival) Before and After Propensity Score Matching in the Eastern and Western Cohorts **eFigure 4.** Adjuvant Chemotherapy Benefit Defined by Maximally Selected Log-Rank Statistics (Y-Axis) in Patients With a Lymph Node Ratio Above the Certain Value (X-Axis) in the Matched Eastern and Western Cohorts **eFigure 5.** Kaplan-Meier Curves for Disease-Free Survival and Disease-Specific Survival Stratified by Lymph Node Ratio in the Matched Eastern Cohort **eFigure 6.** Landmark Analysis of Overall Survival, Disease-Free Survival, and Disease-Specific Survival Stratified by Adjuvant Chemotherapy Cycles in Patients With a Lymph Node Ratio ≥9% in the Matched Eastern Cohort
