# Supplementary material for: Association of Adjuvant Chemotherapy With Overall Survival Among Patients With Locally Advanced Gastric Cancer After Neoadjuvant Chemotherapy
Source: JAMA Netw Open. 2022 Apr 1;5(4):e225557. doi: 10.1001/jamanetworkopen.2022.5557 (PMC8976237; doi:10.1001/jamanetworkopen.2022.5557)
Supplement: Supplement. — eTable 1. Specification and Emulation of a Target Trial eTable 2. Baseline Characteristics of Study Patients Stratified by Receipt of Adjuvant Chemotherapy Before and After Propensity Score Matching in the Western Cohort eTable 3. Univariable and Multivariable Analyses for Overall Survival, Disease-Free Survival, and Disease-Specific Survival in the Matched Eastern Cohort eTable 4. Univariable and Multivariable Analyses for Overall Survival, Disease-Free Survival, and Disease-Specific Survival in Patients With a Lymph Node Ratio <9% in the Matched Eastern Cohort eTable 5. Univariable Analyses for Overall Survival in the Matched Western Cohort eTable 6. Substratified Analysis for Overall Survival, Disease-Free Survival, and Disease-Specific Survival in Patients With a Lymph Node Ratio <9% in the Matched Eastern Cohort eTable 7. Substratified Analysis for Overall Survival, Disease-Free Survival, and Disease-Specific Survival in Patients With a Lymph Node Ratio ≥9% in the Matched Eastern Cohort eFigure 1. Diagram of Study Population in the Eastern (A) and Western (B) Cohorts eFigure 2. The Distribution of Propensity Scores Before and After Matching in the Eastern (A) and Western (B) Cohorts eFigure 3. Kaplan-Meier Curves for Overall Survival (Disease-Free Survival, Disease-Specific Survival) Before and After Propensity Score Matching in the Eastern and Western Cohorts eFigure 4. Adjuvant Chemotherapy Benefit Defined by Maximally Selected Log-Rank Statistics (Y-Axis) in Patients With a Lymph Node Ratio Above the Certain Value (X-Axis) in the Matched Eastern and Western Cohorts eFigure 5. Kaplan-Meier Curves for Disease-Free Survival and Disease-Specific Survival Stratified by Lymph Node Ratio in the Matched Eastern Cohort eFigure 6. Landmark Analysis of Overall Survival, Disease-Free Survival, and Disease-Specific Survival Stratified by Adjuvant Chemotherapy Cycles in Patients With a Lymph Node Ratio ≥9% in the Matched Eastern Cohort [file jamanetwopen-e225557-s001.pdf]

Supplementary Online Content

Lin JX, Tang YH, Lin GJ, et al. Association of adjuvant chemotherapy with overall survival among patients with locally advanced gastric cancer after neoadjuvant chemotherapy. *JAMA Netw Open*. 2022;5(4):e225557. doi:10.1001/jamanetworkopen.2022.5557

eTable 1. Specification and Emulation of a Target Trial

eTable 2. Baseline Characteristics of Study Patients Stratified by Receipt of Adjuvant Chemotherapy Before and After Propensity Score Matching in the Western Cohort

eTable 3. Univariable and Multivariable Analyses for Overall Survival, Disease-Free Survival, and Disease-Specific Survival in the Matched Eastern Cohort

eTable 4. Univariable and Multivariable Analyses for Overall Survival, Disease-Free Survival, and Disease-Specific Survival in Patients With a Lymph Node Ratio <9% in the Matched Eastern Cohort

eTable 5. Univariable Analyses for Overall Survival in the Matched Western Cohort

eTable 6. Substratified Analysis for Overall Survival, Disease-Free Survival, and Disease-Specific Survival in Patients With a Lymph Node Ratio <9% in the Matched Eastern Cohort

eTable 7. Substratified Analysis for Overall Survival, Disease-Free Survival, and Disease-Specific Survival in Patients With a Lymph Node Ratio ≥9% in the Matched Eastern Cohort

eFigure 1. Diagram of Study Population in the Eastern (A) and Western (B) Cohorts

eFigure 2. The Distribution of Propensity Scores Before and After Matching in the Eastern (A) and Western (B) Cohorts

eFigure 3. Kaplan-Meier Curves for Overall Survival (Disease-Free Survival, Disease-Specific Survival) Before and After Propensity Score Matching in the Eastern and Western Cohorts

eFigure 4. Adjuvant Chemotherapy Benefit Defined by Maximally Selected Log-Rank Statistics (Y-Axis) in Patients With a Lymph Node Ratio Above the Certain Value (X-Axis) in the Matched Eastern and Western Cohorts

eFigure 5. Kaplan-Meier Curves for Disease-Free Survival and Disease-Specific Survival Stratified by Lymph Node Ratio in the Matched Eastern Cohort

eFigure 6. Landmark Analysis of Overall Survival, Disease-Free Survival, and Disease-Specific Survival Stratified by Adjuvant Chemotherapy Cycles in Patients With a Lymph Node Ratio ≥9% in the Matched Eastern Cohort

This supplementary material has been provided by the authors to give readers additional information about their work.

© 2022 Lin JX et al. *JAMA Network Open.*

eTable 1. Specification and emulation of a target trial.

Protocol	Target trial specification	Target trial emulation
**Eligibility**	• Aged 18 years or older	Same as for the target trial
**criteria**	• Had histologically confirmed, American Joint Committee on Cancer/Union Internationale Contre le Cancer stage II (T2N1, T1N2, T3N0), IIIA (T3N1, T2N2, T4N0), or IIIB (T3N2) gastric adenocarcinoma with no evidence of metastatic disease	We included patients with locally advanced gastric cancer (cT2-4NxM0)
	• Had D2 surgery and achieved R0 resection	We included patients who underwent neoadjuvant chemotherapy and curative-intent gastrectomy
	• Had a Karnofsky performance status of 70% or more	Same as for the target trial
	• No history of chemotherapy, immunotherapy, radiotherapy, or surgery for gastric cancer	Same as for the target trial
	• Had adequate renal function, hepatic function, and haematological function	Same as for the target trial
**Treatment strategies**	• Patients assigned to the chemotherapy group receive eight 3-week cycles of oral capecitabine (1000 mg/m² twice daily on days 1-14 of each cycle) plus intravenous oxaliplatin (130 mg/m² on day 1 of each cycle).	Patients who underwent 1 or more postoperative adjuvant chemotherapy cycles (5-fluorouracil-based) constituted the adjuvant chemotherapy (AC) group
		We defined the time zero to be 3 months after surgery
**Treatment assignment**	• Patients are randomly assigned to receive adjuvant chemotherapy or surgery alone after surgery, and patients and their physicians will be aware of the assigned treatment strategy	We classified patients according to the strategy that their data were compatible with at baseline and attempted to emulate randomization by adjusting for baseline confounders
**Outcomes**	• 3-year disease-free survival	We defined the primary endpoint as 3-year overall survival because data on recurrence was incomplete in the Western cohort
**Follow-up**	• Survival status was assessed every 3 months for the first 2 years after randomisation, every 6 months for the following year, and yearly thereafter.	Same as for the target trial

© 2022 Lin JX et al. *JAMA Network Open.*

eTable 2. Baseline characteristics of study patients stratified by receipt of adjuvant chemotherapy before and after propensity score matching in the Western cohort.

**Characteristics**	**Overall (n = 109)**	**Before PSM**			**After PSM**	
		**Non-AC (N = 35)**	**AC (N = 74)**	**P value**	**AC (N = 35)**	**P value**
Region, No. (%)				.02		.99
America	88 (80.7)	33 (94.3)	55 (74.3)		34 (97.1)	
Europea	21 (19.3)	2 (5.7)	19 (25.7)		1 (2.9)	
Age, mean (SD), y	61.0 (12.8)	62.7 (12.8)	60.1 (12.7)	.31	58.2 (13.0)	.14
Sex, No. (%)				.74		.10
Male	74 (67.9)	23 (65.7)	51 (68.9)		28 (80.0)	
Female	35 (32.1)	12 (34.3)	23 (31.1)		7 (20.0)	
Body mass index, mean (SD), kg/m^2	23.9 (4.0)	23.6 (4.0)	24.0 (4.0)	.55	24.1 (4.0)	.59
ASA score, No. (%)				.75		.16
1	56 (51.4)	18 (51.4)	38 (51.4)		24 (68.6)	
2	35 (32.1)	10 (28.6)	25 (33.8)		9 (25.7)	
3	18 (16.5)	7 (20.0)	11 (14.9)		2 (5.7)	
Histologic type, No. (%)				.004		.40
Differentiated	43 (39.4)	7 (20.0)	36 (48.6)		10 (28.6)	
Undifferentiated	66 (60.6)	28 (80.0)	38 (51.4)		25 (71.4)	
Type of gastrectomy, No. (%)				.64		.45
Total	65 (59.6)	22 (62.9)	43 (58.1)		25 (71.4)	
Subtotal	44 (40.4)	13 (37.1)	31 (41.9)		10 (28.6)	
Lymph node dissection, No. (%)				.17		.38
D1	20 (18.3)	9 (25.7)	11 (14.9)		6 (17.1)	
D2	89 (81.7)	26 (74.3)	63 (85.1)		29 (82.9)	
Tumor location, No. (%)				.38		.87
Lower 1/3	30 (27.5)	9 (25.7)	21 (28.4)		7 (20.0)	
Middle 1/3	39 (35.8)	10 (28.6)	29 (39.2)		10 (28.6)	
Upper 1/3	21 (28.4)	12 (34.3)	21 (28.4)		15 (42.9)	
Mixed	3 (4.1)	4 (11.4)	3 (4.1)		3 (8.6)	
ypT stage, No. (%)				.10		.64
T0	12 (11.0)	1 (2.9)	11 (14.9)		2 (5.7)	
T1	14 (12.8)	5 (14.3)	9 (12.2)		8 (22.9)	
T2	7 (6.4)	0 (0.0)	7 (9.5)		1 (2.9)	
T3	48 (44.0)	19 (54.3)	29 (39.2)		15 (42.9)	
T4	28 (25.7)	10 (28.6)	18 (24.3)		9 (25.7)	
ypN stage, No. (%)				.87		.94
N0	43 (39.4)	12 (34.3)	31 (41.9)		12 (34.3)	
N1	22 (20.2)	7 (20.0)	15 (20.3)		6 (17.1)	
N2	19 (17.4)	7 (20.0)	12 (16.2)		9 (25.7)	

© 2022 Lin JX et al. *JAMA Network Open.*

N3	25 (22.9)	9 (25.7)	16 (21.6)		8 (22.9)	
Lymph node metastasis, median (IQR)	2 (0-5)	2 (0-7)	1 (0-4)	.75	2 (0-6)	.84
Lymph node harvested, median (IQR)	29 (20-38)	29 (15-38)	29 (20-38)	.66	26 (20-33)	.87
Lymph node ratio, median (IQR)	0.06 (0-0.26)	0.10 (0-0.29)	0.04 (0-0.23)	.68	0.09 (0-0.33)	.80
R status, No. (%)				.66		.99
R0	104 (95.4)	33 (94.3)	71 (95.9)		33 (94.3)	
R1	5 (4.6)	2 (5.7)	3 (4.1)		2 (5.7)	
Postoperative complication, No. (%)				.03		.09
No	88 (80.7)	24 (68.6)	64 (86.5)		30 (85.7)	
Yes	21 (19.3)	11 (31.4)	10 (13.5)		5 (14.3)	

Abbreviations: AC: Adjuvant chemotherapy; PSM: Propensity score match; ASA: American Society of Anesthesiologists; SD: standard deviation; IQR: Interquartile range.

© 2022 Lin JX et al. *JAMA Network Open.*

eTable 3. Univariable and multivariable analyses for overall survival, disease-free survival, and disease-specific survival in the matched Eastern cohort.

Variables	Overall Survival				Disease-free Survival		Disease-specific survival	
	Univariate analysis		Multivariate analysis		Multivariate analysis		Multivariate analysis	
	Hazard Ratio (95%CI)	P value	Hazard Ratio (95%CI)	P value[b]	Hazard Ratio (95%CI)	P value[b]	Hazard Ratio (95%CI)	P value[b]
Age (y)	1.000 (0.982-1.018)	.99						
Sex		.87						
Male	Reference							
Female	0.967 (0.639-1.463)							
Comorbidities		.24						
0-1	Reference							
≥2	1.167 (0.901-1.512)							
Lauren classification		.02		.78		.95		.97
Intestinal	Reference		Reference		Reference		Reference	
Diffuse	1.576 (1.089-2.279)		1.155 (0.780-1.709)		1.040 (0.651-1.662)		1.072 (0.679-1.692)	
Tumor Location		.17						
Lower 1/3	Reference							
Middle 1/3	1.918 (1.103-3.336)							
Upper 1/3	1.328 (0.747-2.361)							
Mixed	2.636 (1.213-5.726)							
ypT stage		<.001		.06		.08		.07
T0-1	Reference		Reference		Reference		Reference	
T2	6.159 (1.536-24.699)		4.940 (1.208-20.195)		2.958 (0.693-12.619)		2.977 (0.707-12.538)	
T3	6.241 (1.434-27.162)		4.525 (1.000-20.467)		4.312 (0.909-20.452)		3.604 (0.762-17.043)	
T4	10.634 (2.630-43.005)		6.091 (1.437-25.828)		4.457 (0.986-20.156)		4.483 (0.996-20.170)	
ypN stage		<.001		.57		.10		.13
N0	Reference		Reference		Reference		Reference	
N1	0.993 (0.516-1.909)		0.729 (0.383-1.389)		1.171 (0.554-2.478)		0.773 (0.371-1.614)	
N2	2.105 (1.299-3.412)		1.280 (0.689-2.379)		1.926 (0.973-3.813)		1.764 (0.879-3.540)	
N3	4.145 (2.605-6.593)		1.240 (0.547-2.809)		1.907 (0.759-4.787)		1.844 (0.719-4.734)	
Lymph node ratio	19.086 (10.132-35.952)	<.001	9.880 (2.850-34.245)	.007	12.038 (3.340-43.380)	.007	10.198 (2.486-41.823)	.02
Lymph node harvested		.68						
<15	Reference							
≥15	0.894 (0.520-1.535)							
Lymphovascular invasion		<.001		.85		.66		.75
No	Reference		Reference		Reference		Reference	
Yes	1.788 (1.272-2.513)		1.091 (0.731-1.628)		1.150 (0.762-1.736)		1.382 (0.722-1.794)	
Neural invasion		.04		.97		.95		.95
No	Reference		Reference		Reference		Reference	
Yes	1.463 (1.020-2.097)		0.980 (0.671-1.431)		0.988 (0.668-1.462)		0.940 (0.623-1.419)	
R status		.005		.66		.95		.56

© 2022 Lin JX et al. *JAMA Network Open.*

R0	Reference		Reference		Reference		Reference	
R1	2.303 (1.295-4.096)		1.290 (0.694-2.399)		1.009 (0.554-1.841)		1.356 (0.686-2.681)	
Tumor regression[a]		.08						
<50%	Reference							
≥50%	0.710 (0.487-1.036)							
Postoperative complication		.79						
No	Reference							
Yes	0.952 (0.660-1.372)							
Adjuvant chemotherapy		.01		.007		.03		.007
No	Reference		Reference		Reference		Reference	
Yes	0.645 (0.455-0.915)		0.565 (0.402-0.794)		0.582 (0.407-0.831)		0.550 (0.384-0.789)	

[a] 37 patients with missed data were excluded from survival analysis.

[b] indicates FDR (false discovery rate) adjusted P value.

Abbreviations: CI: Confidence interval.

© 2022 Lin JX et al. *JAMA Network Open.*

eTable 4. Univariable and multivariable analyses for overall survival, disease-free survival, and disease-specific survival in patients with a lymph node ratio <9% in the matched Eastern cohort.

Variables	Overall Survival				Disease-free Survival		Disease-specific survival	
	Univariate analysis		Multivariate analysis		Multivariate analysis		Multivariate analysis	
	Hazard Ratio (95%CI)	P value	Hazard Ratio (95%CI)	P value[b]	Hazard Ratio (95%CI)	P value[b]	Hazard Ratio (95%CI)	P value[b]
Age (y)	1.011 (0.977-1.046)	.54						
Sex		.57						
Male	Reference							
Female	0.791 (0.356-1.758)							
Comorbidities		.42						
0-1	Reference							
≥2	1.168 (0.804-1.697)							
Lauren classification		.02		.07		.42		.16
Intestinal	Reference		Reference		Reference		Reference	
Diffuse	2.110 (1.137-3.916)		2.113 (1.125-3.971)		1.599 (0.756-3.382)		2.116 (0.954-4.691)	
Tumor Location		.47						
Lower 1/3	Reference							
Middle 1/3	2.418 (0.945-6.182)							
Upper 1/3	0.900 (0.331-2.446)							
Mixed	3.809 (1.353-10.723)							
ypT stage		.03		.09		.16		.16
T0-1	Reference		Reference		Reference		Reference	
T2-4	8.629 (1.251-59.500)		9.246 (1.315-65.029)		6.917 (0.927-51.612)		6.575 (0.905-47.746)	
ypN stage		.72						
N0	Reference							
N+	0.873 (0.419-1.823)							
Lymphovascular invasion		.97						
No	Reference							
Yes	1.012 (0.504-2.032)							
Neural invasion		.12						
No	Reference							
Yes	1.621 (0.883-2.988)							
R status		.47						
R0	Reference							
R1	1.633 (0.434-6.138)							
Tumor regression[a]		.54						
<50%	Reference							
≥50%	0.790 (0.372-1.676)							
Postoperative complication		.02		.07		.06		.34

© 2022 Lin JX et al. *JAMA Network Open.*

No	Reference		Reference		Reference		Reference	
Yes	2.129 (1.140-3.974)		2.064 (1.122-3.799)		2.603 (1.248-5.429)		1.752 (0.812-3.779)	
Adjuvant chemotherapy		.29		.61		.88		.86
No	Reference		Reference		Reference		Reference	
Yes	0.721 (0.392-1.326)		0.768 (0.413-1.428)		0.878 (0.404-1.909)		0.865 (0.401-1.867)	

[a] 17 patients with missed data were excluded from survival analysis.

[b] indicates FDR (false discovery rate) adjusted P value.

Abbreviations: CI: Confidence interval.

© 2022 Lin JX et al. *JAMA Network Open.*

eTable 5. Univariable analyses for overall survival in the matched Western cohort.

Variables	LNR <9%		LNR ≥9%	
	Hazard Ratio (95% CI)	P value	Hazard Ratio (95% CI)	P value
Age (y)	1.022 (0.972-1.074)	.40	0.977 (0.949-1.006)	.13
Sex		.11		.11
Male	Reference		Reference	
Female	2.402 (0.817-7.062)		2.121 (0.849-5.346)	
Body mass index (kg/m^2)	1.018 (0.900-1.151)	.77	0.951 (0.873-1.037)	.26
ASA score		.87		.87
1	Reference		Reference	
≥2	0.916 (0.323-2.595)		1.075 (0.461-2.506)	
Histologic type		.16		.66
Differentiated	Reference		Reference	
Undifferentiated	4.558 (0.543-38.275)		1.278 (0.432-3.781)	
Lymph node dissection		.06		.61
D1	Reference		Reference	
D2	0.346 (0.115-1.043)		1.206 (0.588-2.471)	
Tumor Location		.75		.91
Lower 1/3	Reference		Reference	
Middle 1/3	1.441 (0.543-3.828)		0.670 (0.188-2.391)	
Upper 1/3	0.782 (0.188-3.257)		0.614 (0.150-2.509)	
Mixed	34.832 (5.051-240.223)		1.369 (0.292-6.413)	
ypT stage		.20		.001
T0-2	Reference		Reference	
T3	2.849 (0.838-9.685)		9.802 (1.618-59.380)	
T4	1.351 (0.110-16.623)		11.554 (1.883-70.890)	
Postoperative complication		.83		.90
No	Reference		Reference	
Yes	0.866 (0.239-3.136)		1.060 (0.434-2.586)	
Adjuvant chemotherapy		.91		.02
No	Reference		Reference	
Yes	0.950 (0.380-2.377)		0.429 (0.207-0.891)	

Abbreviations: LNR: Lymph node ratio; ASA: American Society of Anesthesiologists; CI: Confidence interval.

© 2022 Lin JX et al. *JAMA Network Open.*

eTable 6. Substratified analysis for overall survival in patients with a lymph node ratio <9% in the matched Eastern cohort.

Variables	Subgroup	Hazard Ratio (95% CI)	P value	FDR adjusted P for interaction
Age, yrs	<60	0.656 (0.284-1.515)	.32	.97
	≥60	0.786 (0.346-1.789)	.57	
Sex	Male	0.925 (0.476-1.800)	.82	.31
	Female	0.275 (0.069-1.097)	.07	
Comorbidities	0-1	0.624 (0.326-1.191)	.15	.85
	≥2	1.515 (0.276-8.325)	.63	
Lauren classification	Intestinal	0.351 (0.099-1.247)	.11	.92
	Diffuse	1.077 (0.520-2.228)	.84	
Tumor Location	Lower 1/3	3.185 (0.494-20.538)	.22	.26
	Middle 1/3	0.909 (0.376-2.198)	.83	
	Upper 1/3	0.646 (0.186-2.239)	.49	
	Mixed	0.185 (0.027-1.258)	.08	
ypT stage	T0-1	NE	NE	.78
	T2-4	0.776 (0.422-1.427)	.42	
ypN stage	N0	0.793 (0.381-1.653)	.54	.61
	N+	0.571 (0.175-1.864)	.35	
Lymph node harvested	<15	0.663 (0.144-3.062)	.60	.97
	≥15	0.731 (0.380-1.406)	.35	
Lymphovascular invasion	No	0.789 (0.371-1.676)	.54	.66
	Yes	0.610 (0.200-1.864)	.39	
Neural invasion	No	1.056 (0.462-2.416)	.90	.93
	Yes	0.458 (0.179-1.175)	.10	
R status	R0	0.646 (0.345-1.211)	.17	.16
	R1	NE	NE	
Tumor regression[a]	<50%	0.495 (0.112-2.181)	.35	.93
	≥50%	1.060 (0.465-2.414)	.89	
Postoperative complication	No	0.563 (0.262-1.210)	.14	.13
	Yes	1.153 (0.394-3.371)	.80	

[a] 17 patients with missed data were excluded from survival analysis.

Abbreviations: CI: Confidence interval; FDR: False discovery rate; NE: Not evaluable.

© 2022 Lin JX et al. *JAMA Network Open.*

eTable 7. Substratified analysis for overall survival in patients with a lymph node ratio ≥9% in the matched Eastern cohort.

**Variables**	**Subgroup**	**Hazard Ratio (95% CI)**	**P value**	**FDR adjusted P for interaction**
Age, yrs	<60	0.514 (0.255-1.036)	.06	.12
	≥60	0.407 (0.228-0.725)	.002	
Sex	Male	0.437 (0.267-0.716)	.001	.93
	Female	0.513 (0.232-1.135)	.10	
Comorbidities	0-1	0.420 (0.257-0.688)	<.001	.61
	≥2	0.787 (0.302-2.048)	.62	
Lauren classification	Intestinal	0.452 (0.192-1.065)	.07	.85
	Diffuse	0.442 (0.26-0.751)	.003	
Tumor Location	Lower 1/3	0.406 (0.137-1.204)	.10	.65
	Middle 1/3	0.455 (0.207-1.000)	.05	
	Upper 1/3	0.374 (0.196-0.713)	.003	
	Mixed	NE	NE	
ypT stage	T1-2	1.070 (0.383-2.995)	.90	.03
	T3	0.177 (0.078-0.402)	<.001	
	T4	0.492 (0.276-0.879)	.02	
ypN stage	N1	0.511 (0.117-2.224)	.37	.23
	N2	0.363 (0.191-0.689)	.002	
	N3	0.589 (0.330-1.053)	.07	
Lymph node harvested	<15	0.326 (0.070-1.513)	.15	.47
	≥15	0.466 (0.295-0.735)	.001	
Lymphovascular invasion	No	0.554 (0.271-1.134)	.11	.73
	Yes	0.369 (0.207-0.659)	<.001	
Neural invasion	No	0.388 (0.217-0.694)	.001	.79
	Yes	0.585 (0.298-1.147)	.12	
R status	R0	0.448 (0.284-0.706)	<.001	.16
	R1	0.579 (0.155-2.158)	.42	
Tumor regression[a]	<50%	0.413 (0.199-0.857)	.02	.34
	≥50%	0.532 (0.300-0.941)	.03	
Postoperative complication	No	0.426 (0.252-0.720)	.001	.19
	Yes	0.521 (0.235-1.155)	.11	

[a] 20 patients with missed data were excluded from survival analysis.

Abbreviations: CI: Confidence interval; FDR: False discovery rate; NE: Not evaluable.

© 2022 Lin JX et al. *JAMA Network Open.*

eFigure 1. Diagram of study population in the Eastern (A) and Western (B) cohorts.

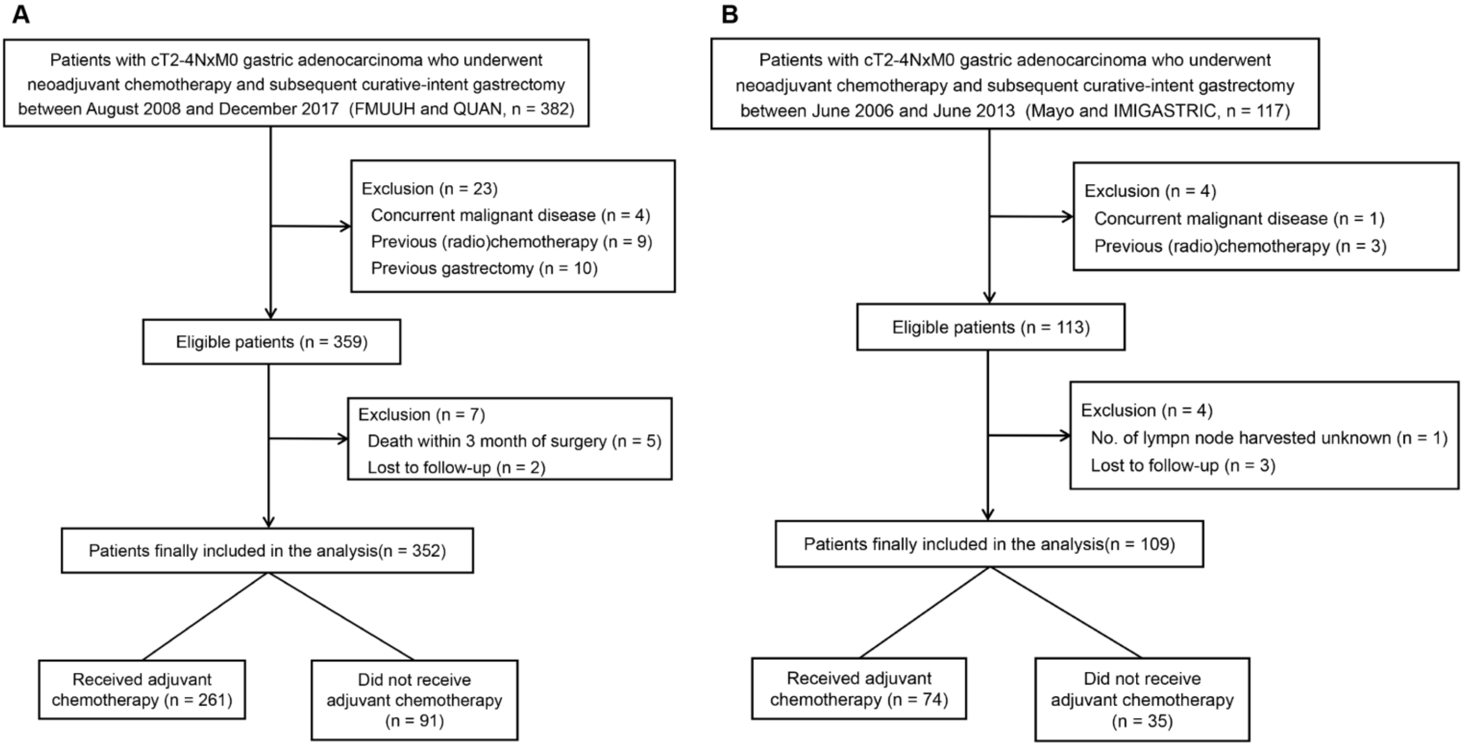

© 2022 Lin JX et al. *JAMA Network Open.*

eFigure 2. The distribution of propensity scores before and after matching in the Eastern (A) and Western (B) cohorts.

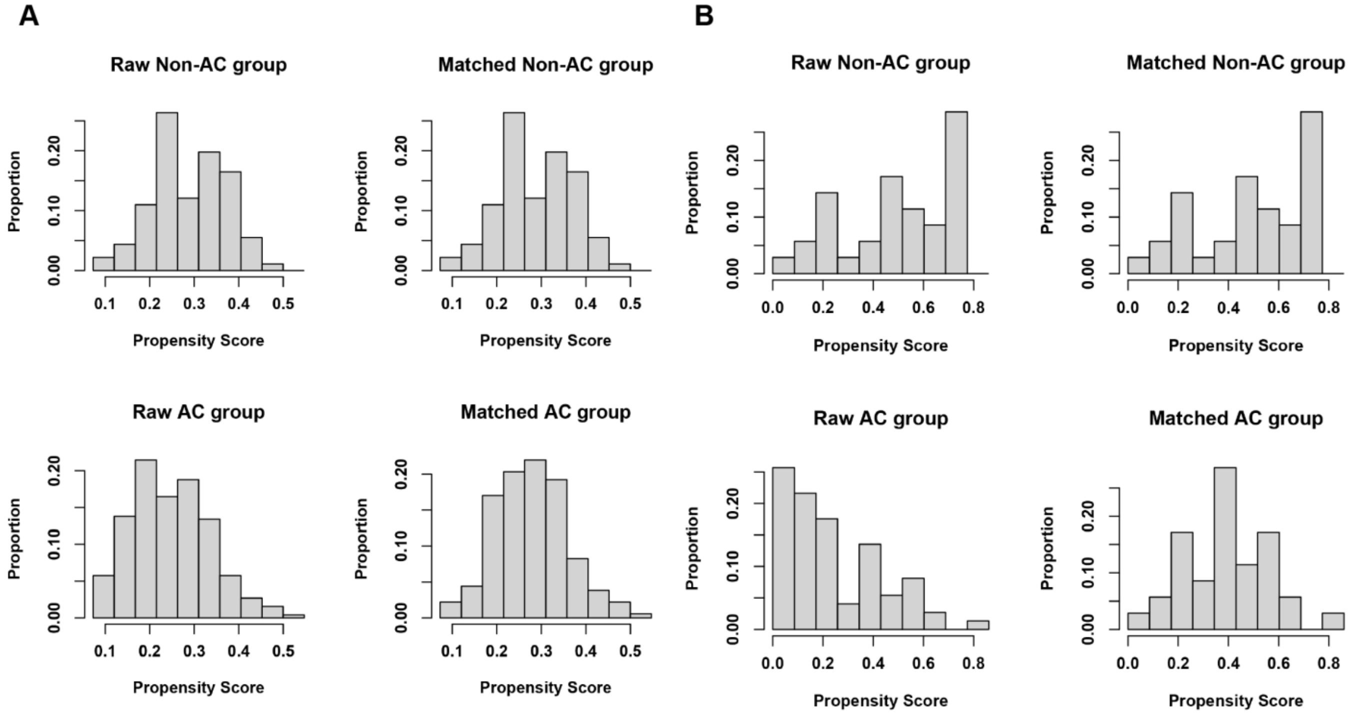

© 2022 Lin JX et al. *JAMA Network Open.*

eFigure 3. Kaplan-Meier curves for overall survival (disease-free survival, disease-specific survival) before and after propensity score matching in the Eastern and Western cohorts. We calculated the P values using the log-rank test. The time zero was set as 3 months after surgery.

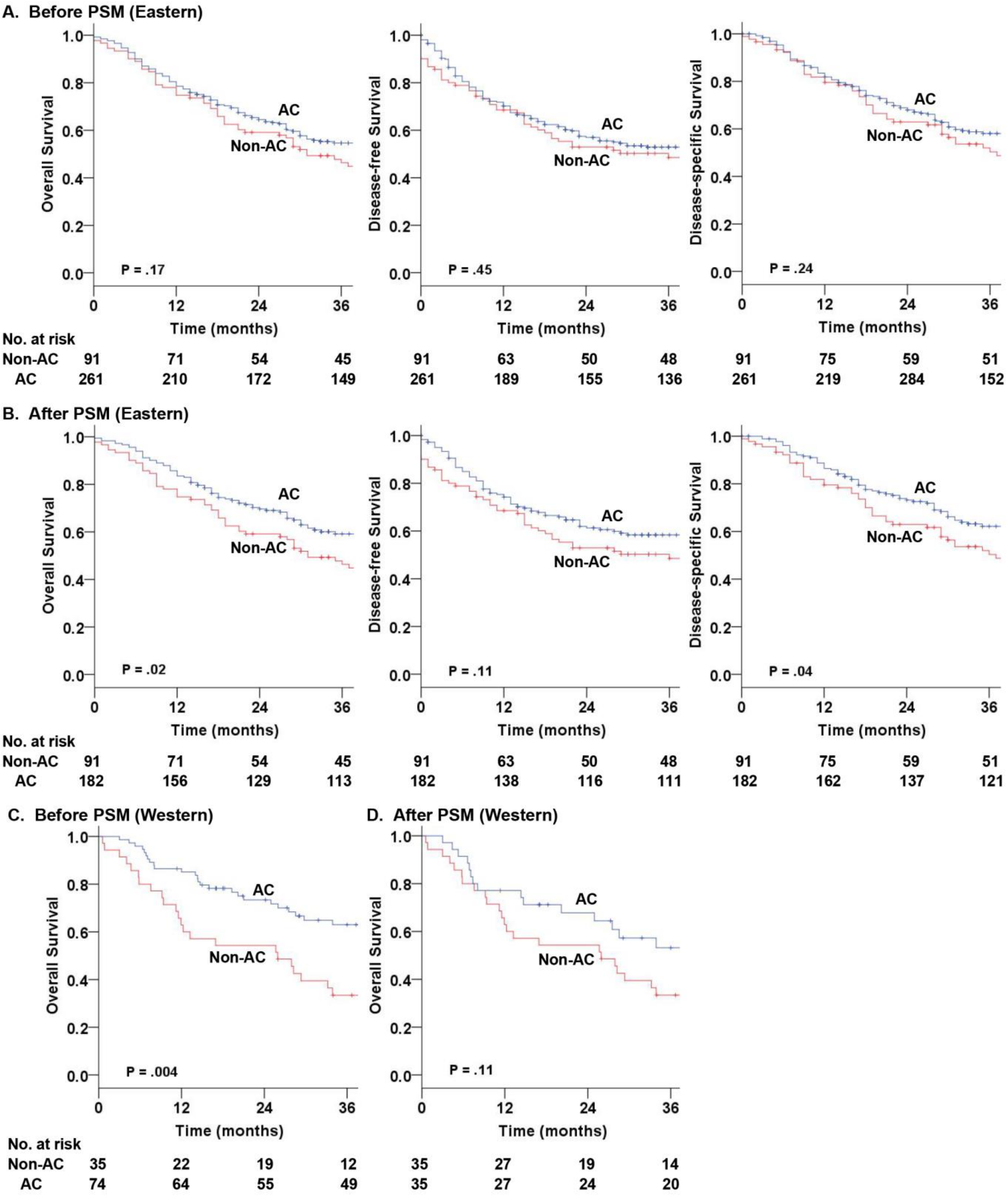

© 2022 Lin JX et al. *JAMA Network Open.*

eFigure 4. Adjuvant chemotherapy benefit defined by maximally selected log-rank statistics (Y-axis) in patients with a lymph node ratio above the certain value (X-axis) in the matched Eastern and Western cohorts.

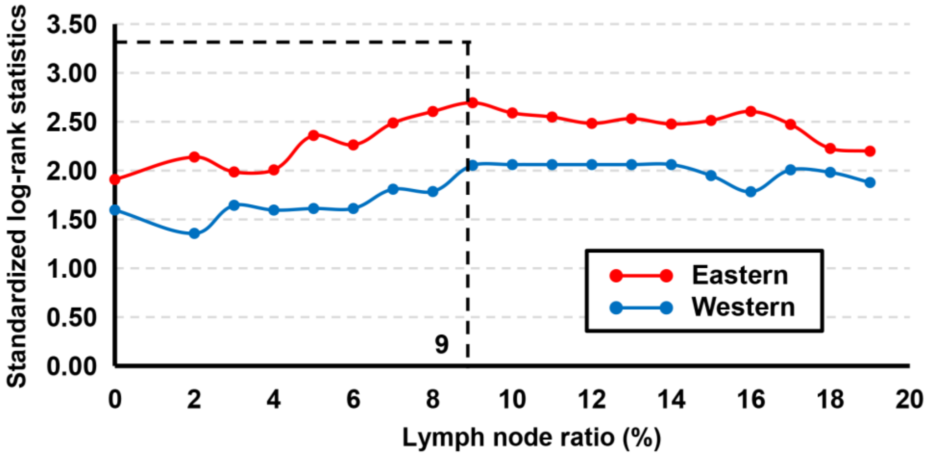

© 2022 Lin JX et al. *JAMA Network Open.*

eFigure 5. Kaplan-Meier curves for disease-free survival and disease-specific survival stratified by lymph node ratio in the matched Eastern cohort. The time zero was set as 3 months after surgery.

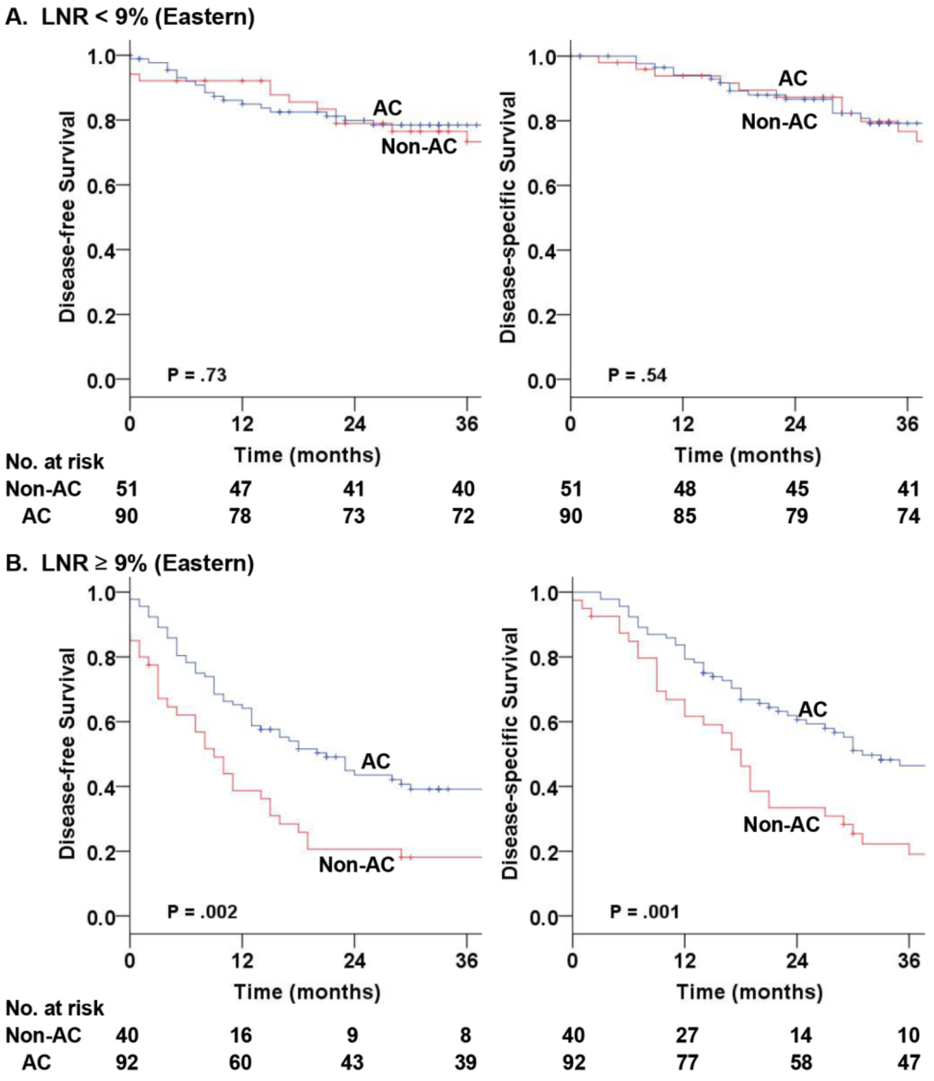

© 2022 Lin JX et al. *JAMA Network Open.*

eFigure 6. Landmark analysis of overall survival, disease-free survival, and disease-specific survival stratified by adjuvant chemotherapy cycles in patients with a lymph node ratio ≥9% in the matched Eastern cohort. Patients who experienced death or disease recurrence within 6 months, 9 months after surgery were excluded from the 6-month (A) and 9-month (B) landmark analysis, with sample sizes of 108 and 98, respectively. The time zero was set as 6 months (A) and 9 months (B) after surgery.

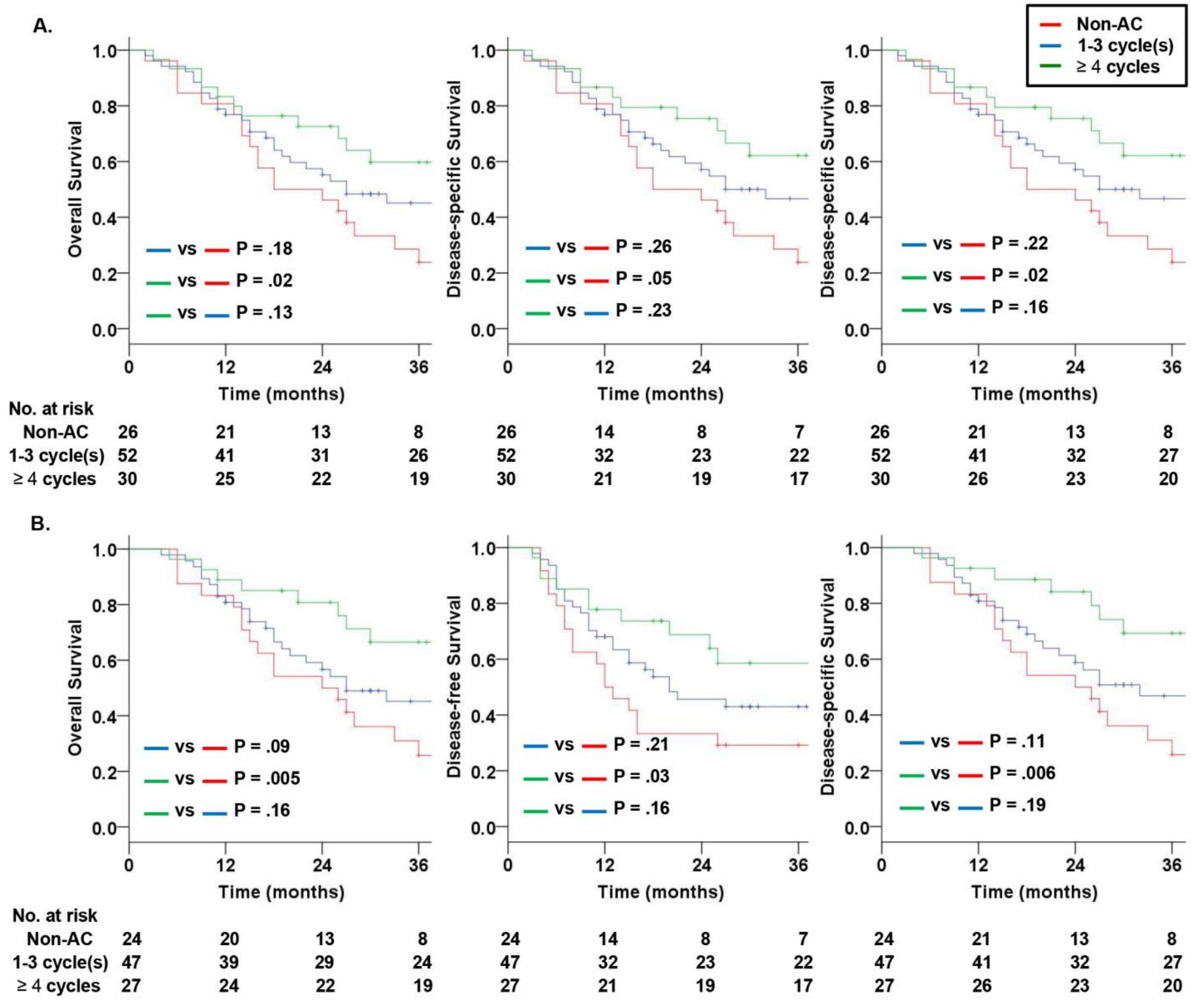

© 2022 Lin JX et al. *JAMA Network Open.*
